# [18F]FDG PET/CT-based response assessment of stage IV non-small cell lung cancer treated with paclitaxel-carboplatin-bevacizumab with or without nitroglycerin patches

**DOI:** 10.1007/s00259-016-3498-y

**Published:** 2016-09-06

**Authors:** Evelyn E. C. de Jong, Wouter van Elmpt, Ralph T. H. Leijenaar, Otto S. Hoekstra, Harry J. M. Groen, Egbert F. Smit, Ronald Boellaard, Vincent van der Noort, Esther G. C. Troost, Philippe Lambin, Anne-Marie C. Dingemans

**Affiliations:** 1 Department of Radiation Oncology (MAASTRO), GROW-School for Oncology and Developmental Biology, Maastricht University Medical Centre, Maastricht, Netherlands; 2 Department of Nuclear Medicine & PET Research, VU University Medical Center, Amsterdam, Netherlands; 3 Department of Pulmonary Diseases, University of Groningen and University Medical Center Groningen, Groningen, Netherlands; 4 Department of Pulmonary Diseases, VU University Medical Center, Amsterdam, Netherlands; 5 Department of Thoracic Oncology, The Netherlands Cancer Institute-Antoni van Leeuwenhoek Hospital, Amsterdam, Netherlands; 6 Department of Nuclear Medicine and Molecular Imaging, University Medical Center Groningen, Groningen, Netherlands; 7 Department of Biometrics, The Netherlands Cancer Institute-Antoni van Leeuwenhoek Hospital, Amsterdam, Netherlands; 8 Institute of Radiooncology, Helmholtz-Zentrum Dresden-Rossendorf, Dresden, Germany; 9 Department of Radiotherapy and Radiation Oncology, Medical Faculty and University Hospital Carl Gustav Carus of Technische Universität Dresden, Dresden, Germany; 10 Department of Pulmonology, GROW-School for Oncology and Developmental Biology, Maastricht University Medical Centre, Maastricht, Netherlands

**Keywords:** [18F]FDG PET/CT, Response assessment, Nitroglycerin, Bevacizumab, Stage IV NSCLC

## Abstract

**Purpose:**

Nitroglycerin (NTG) is a vasodilating drug, which increases tumor blood flow and consequently decreases hypoxia. Therefore, changes in [18F] fluorodeoxyglucose positron emission tomography ([18F]FDG PET) uptake pattern may occur. In this analysis, we investigated the feasibility of [18F]FDG PET for response assessment to paclitaxel-carboplatin-bevacizumab (PCB) treatment with and without NTG patches. And we compared the [18F]FDG PET response assessment to RECIST response assessment and survival.

**Methods:**

A total of 223 stage IV non-small cell lung cancer (NSCLC) patients were included in a phase II study (NCT01171170) randomizing between PCB treatment with or without NTG patches. For 60 participating patients, a baseline and a second [18F]FDG PET/computed tomography (CT) scan, performed between day 22 and 24 after the start of treatment, were available. Tumor response was defined as a 30 % decrease in CT and PET parameters, and was compared to RECIST response at week 6. The predictive value of these assessments for progression free survival (PFS) and overall survival (OS) was assessed with and without NTG.

**Results:**

A 30 % decrease in SUVpeak assessment identified more patients as responders compared to a 30 % decrease in CT diameter assessment (73 % vs. 18 %), however, this was not correlated to OS (SUVpeak30 *p* = 0.833; CTdiameter30 *p* = 0.557). Changes in PET parameters between the baseline and the second scan were not significantly different for the NTG group compared to the control group (*p* value range 0.159–0.634). The CT-based (part of the [18F]FDG PET/CT) parameters showed a significant difference between the baseline and the second scan for the NTG group compared to the control group (CT diameter decrease of 7 ± 23 % vs. 19 ± 14 %, *p* = 0.016, respectively).

**Conclusions:**

The decrease in tumoral FDG uptake in advanced NSCLC patients treated with chemotherapy with and without NTG did not differ between both treatment arms. Early PET-based response assessment showed more tumor responders than CT-based response assessment (part of the [18F]FDG PET/CT); this was not correlated to survival. This might be due to timing of the [18F]FDG PET shortly after the bevacizumab infusion.

## Introduction

Molecular imaging with [18F] fluorodeoxoyglucose positron emission tomography ([18F]FDG PET) has an established role in the staging of patients with non-small cell lung cancer (NSCLC). In addition, an increasing number of studies have shown that [18F]FDG PET is useful for early response assessment in NSCLC patients treated with cytotoxic chemotherapy [1–4].

Tumor hypoxia is a common phenomenon in lung cancer and it is related to poor prognosis due to treatment resistance [5–11]. Preclinical studies have shown that nitric oxide (NO)-donating drugs increase blood flow and thereby decrease hypoxia [12]. Nitroglycerin (NTG), a vasodilator, is such a drug. By increasing the tumor blood flow, NTG consequently augments antitumor drug delivery and inhibits hypoxia inducible factor (HIF-1α) [13]. In preclinical models, administration of low doses of NTG, at least partially, reverses the hypoxia-induced resistance to anticancer drugs [14].

Yasuda et al. [15] showed that the combination of platinum-based chemotherapy and NTG improves overall survival (OS) in patients with stage IIIb/IV NSCLC. However, two recent randomized studies, including the Dutch NVALT12 study, could not confirm these results and no clinical effect was observed by the addition of NTG [16, 17].

Negative correlation between perfusion computed tomography (CT) and hypoxia PET on a population basis were also described in literature [18], suggesting that hypoxia is negatively correlated to tumor blood flow. Consequently, if treatment with NTG improved tumor perfusion, this could translate into a change in FDG uptake [13]. This concept was tested in the context of the randomized NVALT12 study that sought to investigate whether the addition of NTG to first-line paclitaxel-carboplatin-bevacizumab (PCB) chemotherapy would improve progression free survival (PFS).

In clinical practice, tumor response assessment is based on changes in tumor size, according to response evaluation criteria in solid tumors (RECIST) at week 6 [19, 20]. However, response monitoring is complex because the tumor has to change significantly in size and shape before a response is reliably detected by CT [21, 22]. This leads to an underestimation of the efficacy of cytostatic therapeutic agents that stabilize the disease, in contrast to conventional cytotoxic drugs, which induce shrinkage of tumor dimensions in the case of tumor response [19]. Metabolic changes, measured by [18F]FDG PET, will occur earlier than changes in size and may, therefore, be used for early treatment response assessment. A decrease in metabolic activity of the primary tumor after one cycle of chemotherapy treatment is predictive for better outcome [1, 18, 23, 24].

In this paper, we investigated the feasibility of [18F]FDG PET for response assessment to PCB treatment with and without NTG patches. Furthermore, we compared the [18F]FDG PET response with both the commonly used RECIST and survival.

## Materials and methods

### Patient characteristics

**Fig. 1:**
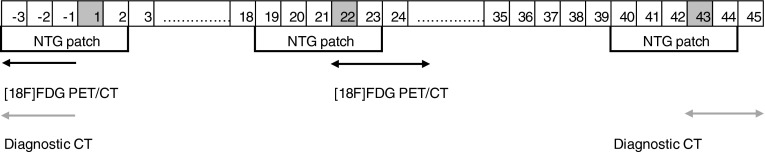
NVALT12 trial timeline. At day one of the 21-day cycle, the paclitaxel-carboplatin-bevacizumab therapy is administered (*grey square*). The patients in the experimental arm wear the nitroglycerin (NTG) patch from day −3 to +2. The baseline [18F]FDG PET/CT is performed before the start of chemotherapy and the second [18F]FDG PET/CT is performed between day 22 and 24 (*black arrow*). The baseline diagnostic CT is performed before the start of chemotherapy and repeated after every two cycles of chemotherapy (*grey arrow*)

In the multicentric NVALT12 trial (NCT01171170), 223 patients with metastatic non-squamous NSCLC were randomized between PCB with or without NTG (see [17] for patient inclusion criteria and treatment specifications) with the primary endpoint of that trial being PFS. Response was assessed every two cycles by the local investigator according to RECIST 1.1 based on CT imaging [20]. In patients undergoing [18F]FDG PET/CT at baseline as part of the standard work-up (median number of days between baseline scan and start treatment 17 days; range 73 days before treatment to 1 scan performed 1 day after the start of treatment), the protocol pre-specified a second [18F]FDG PET/CT between day 22 and 24 (after second chemotherapy infusion and with NTG application for patients in the experimental group; Fig. 1). To include more patients (17) in the analysis presented here, scans acquired with a time interval between the first chemotherapy and the second [18F]FDG PET/CT scan less than 35 days were accepted. This study was approved by the medical ethical committee and all patients provided informed written consent prior to any study handling.

### Scan protocol

Injected [18F]FDG activity depended on individual patient and scanner characteristics, following the Netherlands protocol for standardization of [18F]FDG whole-body PET studies in multi-center trials (NEDPAS) [25], which was the precursor of the EANM guidelines, and images were reconstructed to institutional standards. Typically, a low-dose CT scan as part of the [18F]FDG PET/CT was made, according to institutional standards, and used for attenuation correction. Due to variations between the institutes, for quality control purposes, a spherical volume of interest (VOI) with a diameter of 3 cm was delineated in the right lobe of the liver [26]. This measurement was used as quality index and scans with a mean standardized uptake value (SUV) of the liver below 1.3 or above 3.0 were excluded from further analysis [27].

### Early prediction of survival

The primary tumor was manually delineated by experienced radiation oncologists using a treatment planning system (Eclipse Version 11.0, Varian Medical Systems, Inc.) and used as the region of interest (ROI). A standard delineation protocol was used, which included fixed window/level settings of CT (lung: 1700/-300; mediastinum: 600/40). Patients without a measurable primary tumor on the baseline [18F]FDG PET/CT scan were excluded from analysis.

The maximum standardized uptake value (SUVmax), mean SUV (SUVmean), peak SUV (SUVpeak; mean uptake in a sphere with a diameter of 1.2 cm [21]), total lesion glycolysis (TLG; TLG was defined by SUVmean multiplied by the tumor volume), maximal CT diameter, and CT volume (number of voxels within the delineated ROI multiplied by the voxel size) were calculated in our institute on the [18F]FDG PET/CT scan (Matlab R2013a, The Mathworks, Natrick, MA, USA) using an adapted version of CERR (Computational Environment for Radiotherapy Research) extended with in-house developed Radiomics image analysis software to extract imaging features [28, 29]. Early metabolic response was defined using relative changes in [18F]FDG PET uptake parameters of the primary tumor expressed as a percentage change from baseline. Patients were grouped according to a 30 % decrease in CT and PET parameters in the primary tumor ROI of the [18F]FDG PET/CT scan [26, 30, 31]. For the PET response assessment, SUVpeak was used and for the CT response assessment, CT diameter (CT was part of the [18F]FDG PET/CT) was used [26, 30]. The RECIST analysis performed during week 6 by the local investigators was used in the analysis to separate patients into responders and non-responders. The 30 % CT and PET response assessments, performed after 3 weeks of therapy, were compared against the RECIST response assessment performed during week 6 by a specificity and sensitivity analysis.

### Statistics

Since normality checks suggested an abnormal distribution for the changes in CT and PET parameters from baseline, non-parametric tests were used for the analysis of these variables. Comparison of the mean changes in CT and PET parameters from baseline for responders vs. non-responders was carried out by an independent samples Mann–Whitney *U* test. PFS was defined as the interval from randomization to progressive disease or death, whichever occurred first, and OS was defined as the interval from randomization to death from any cause. Differences in PFS and OS were investigated using Cox regression. For calculating the hazard ratio (HR), the different response assessment criteria were used, as a binary variable. To compare CT diameter and SUVmax response with survival, in the waterfall plots a survival cut-off of 6 months was used. This is the median PFS of the combined group (NTG group combined with control group). Statistical tests were based on a two-sided significance level, and the level of significance was set at 0.05. All statistics were performed in SPSS v.21 (IBM Corp. Released 2012, IBM SPSS Statistics for Windows, Version 21.0, Armonk, NY, USA).

## Results

### Patients

**Fig. 2:**
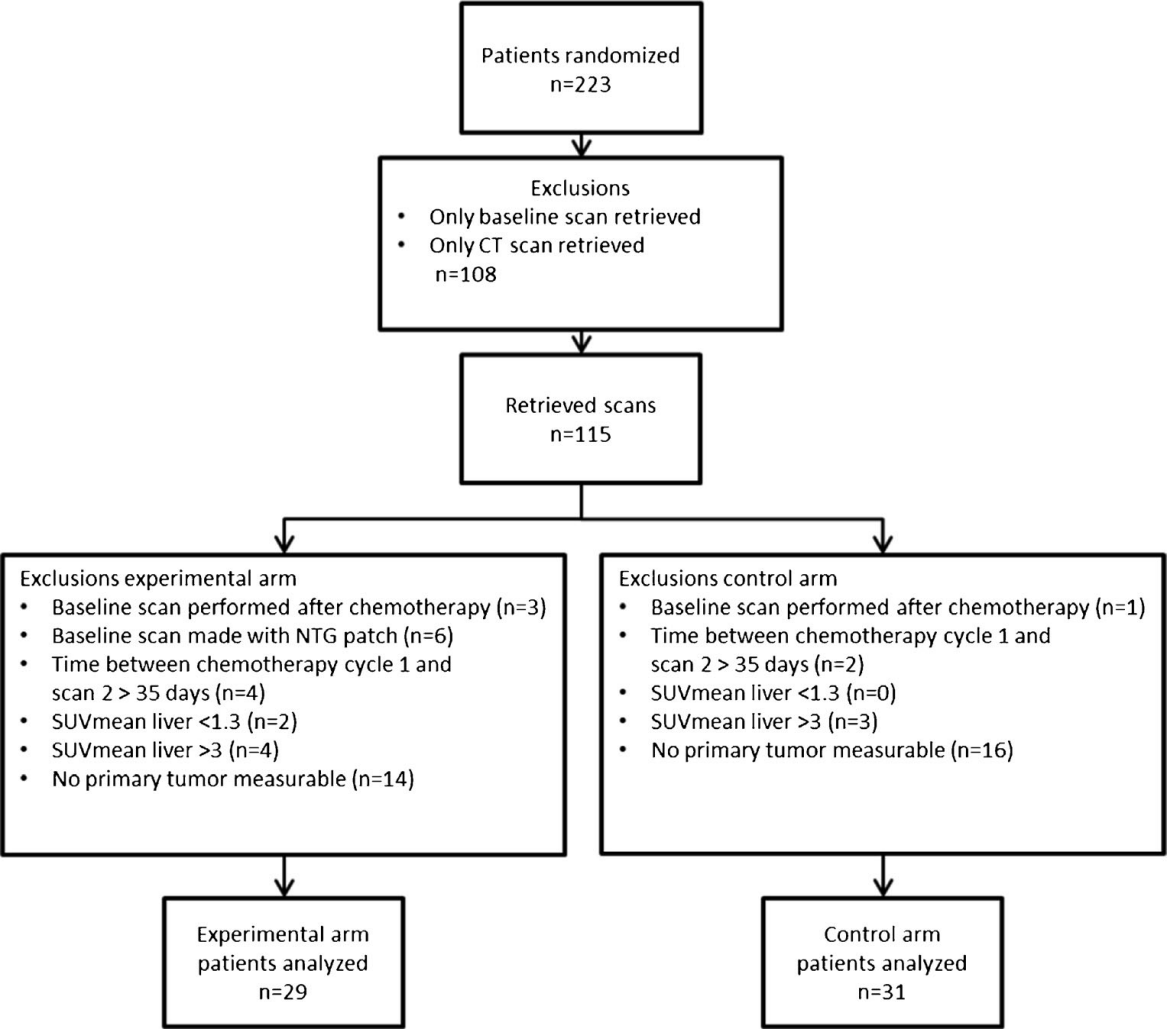
CONSORT diagram. SUV: standardized uptake value

87 out of the 223 included patients in the randomized phase II study had two [18F]FDG PET/CT scans available with a measurable primary tumor; however, 27 patients were subsequently excluded for analysis due to poor image quality (see methods). Hence, 60 patients (characteristics in Table 1) had two evaluable consecutive [18F]FDG PET/CT scans (Fig. 2) with a median interval of 42 days. PFS and OS were similar for patients treated with PCB and PCB + NTG (Table 1).

**Table 1:** Patient characteristics

		Control	Experimental
Patients analyzed		31	29
Gender	Male	15	15
	Female	16	14
Age (mean, range)	[years]	59 (39–73)	59 (45–77)
WHO-PS	0	20 (65 %)	10 (34 %)
	1	10 (32 %)	18 (62 %)
	2	1 (3 %)	1 (3 %)
Smoker	Current	13 (42 %)	14 (48 %)
	Ex	14 (45 %)	12 (41 %)
	Never	4 (13 %)	3 (11 %)
Histology	Adeno	27 (86 %)	24 (83 %)
	Large cell	2 (7 %)	1 (3 %)
	Other	2 (7 %)	4 (14 %)
Survival (median, range)	PFS [months]	7 (3–25)	4 (1–11)
	OS [months]	13 (4–33)	9 (2–29)
RECIST response (week 6)	Complete response	0 (0 %)	0 (0 %)
	Partial response	9 (29 %)	5 (17 %)
	Stable disease	20 (65 %)	17 (59 %)
	Progressive disease	2 (6 %)	7 (24 %)
Baseline (mean, range)	CT diameter [cm]	6.8 (2.5–12.1)	6.7 (2.4–16.4)
	CT volume [cm^3^]	101.5 (4.4–474.5)	89.8 (3.0–468.8)
	SUVmax	13.5 (3.4–28.9)	14.5 (3.6–44.6)
	SUVmean	5.7 (2.5–11.8)	6.2 (2.1–22.3)
	SUVpeak	11.0 (3.1–25.3)	12.1 (2.6–37.9)
	TLG [SUV*cm^3^]	655.0 (23.4–4288.8)	638.4 (8.2–3467.2)

*WHO-PS* World Health Organization performance status, *PFS* progression-free survival, *OS* overall survival, *RECIST* response evaluation criteria in solid tumors, *SUV* standardized uptake value, *TLG* total lesion glycolysis

### Image characteristics

#### Experimental vs. control arm

**Fig. 3:**
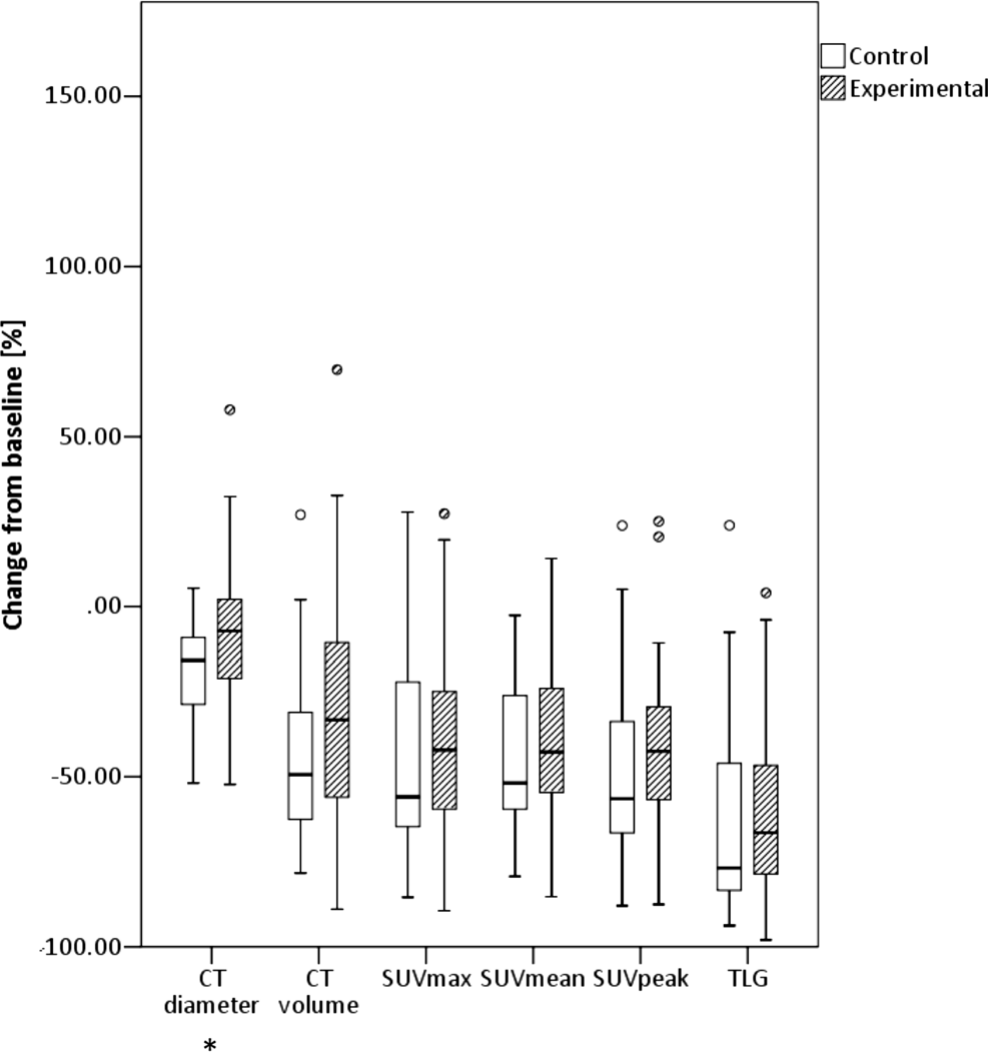
Mean values and standard deviations for the CT- and PET-derived image parameters for the experimental arm and the control arm. *p* values of the independent samples Mann–Whitney *U* test of the mean change from baseline of the control arm vs. the mean change from baseline of the experimental arm (*significantly different for the experimental arm compared to the control arm with a significance level of 5 %). SUV: standardized uptake value; TLG: total lesion glycolysis

The mean decrease in SUVmax between the 31 patients treated with PCB (46 ± 27 %) and the 29 patients treated with PCB + NTG (42 ± 29 %) was not statistically significantly different (*p* = 0.510). The other PET parameters (SUVmean, SUVpeak and TLG) showed on average > 40 % decrease from baseline, but this was also not statistically significantly different between the experimental arm and the control arm (Fig. 3). Although for CT, part of the [18F]FDG PET/CT, in the control arm, the CT diameter decreased significantly more than in the experimental arm (19 ± 14 % vs. 7 ± 23 %; *p* = 0.028).

#### Early prediction of survival

According to the 30 % PET criteria, 74 % of patients in the control arm and 72 % of the patients in the experimental arm showed response after 3 weeks (median time interval 42 days). According to the 30 % CT criteria, 26 % of the patients in the control arm and 10 % of the patients in the experimental arm had a response. According to the RECIST analysis performed after 2 cycles (median time interval 56 days) by the local investigator, 29 % of the patients in the control arm had a response and 17 % of the patients in the experimental arm had a response (Table 1).

The predictive value of the 30 % CT-based and 30 % PET-based response assessments performed after 3 weeks (on the primary tumor) was assessed for response according to RECIST after 2 cycles (Table 2). The 30 % PET-based response assessment had a higher sensitivity compared to the 30 % CT-based response assessment but a lower specificity (Table 2).

**Table 2:** Comparison of 30 % CT-based and 30 % PET-based response assessment performed after 3 weeks with the RECIST response assessment of week 6

	RECIST	RECIST	Total
	Responder	Non-responder	
CT diameter decrease >30 %	9 (15 %)	2 (3 %)	11 (18 %)
CT diameter decrease <30 %	17 (28 %)	32 (54 %)	49 (82 %)
	26 (43 %)	34 (57 %)	60 (100 %)
	Sensitivity = 35 %	Specificity = 94 %	
SUVpeak decrease >30 %	23 (38 %)	21 (35 %)	44 (73 %)
SUVpeak decrease <30 %	3 (5 %)	13 (22 %)	16 (27 %)
	26 (43 %)	34 (57 %)	60 (100 %)
	Sensitivity = 88 %	Specificity = 38 %	

*SUV* standardized uptake value, *RECIST* response evaluation criteria in solid tumors

The 30 % CT-based and 30 % PET-based response assessments were for neither of the arms predictive for PFS nor OS (Table 3).

**Table 3:** The hazard ratios (HR) for 30 % PET- and CT-based response assessment with 95 % confidence interval and corresponding *p* values for OS and PFS are shown per parameter

SUV parameter		PFS		OS	
		HR (95 % CI)	*p* value	HR (95 % CI)	*p* value
30 % response assessment	SUVmax	1.048 (0.591–1.858)	0.874	1.025 (0.572–1.837)	0.934
	SUVmean	0.941 (0.527–1.680)	0.838	0.901 (0.501–1.619)	0.726
	SUVpeak	0.929 (0.514–1.679)	0.807	0.938 (0.515–1.706)	0.833
	TLG	0.706 (0.355–1.406)	0.323	1.511 (0.722–3.160)	0.273
	CTvolume	1.073 (0.617–1.866)	0.802	1.338 (0.740–2.419)	0.335
	CTdiameter	0.718 (0.370–1.390)	0.325	0.805 (0.390–1.662)	0.557

*SUV* standardized uptake value, *TLG* total lesion glycolysis, *PFS* progression free survival, *OS* overall survival, *HR* hazard ratio, *CI* confidence interval

**Fig. 4:**
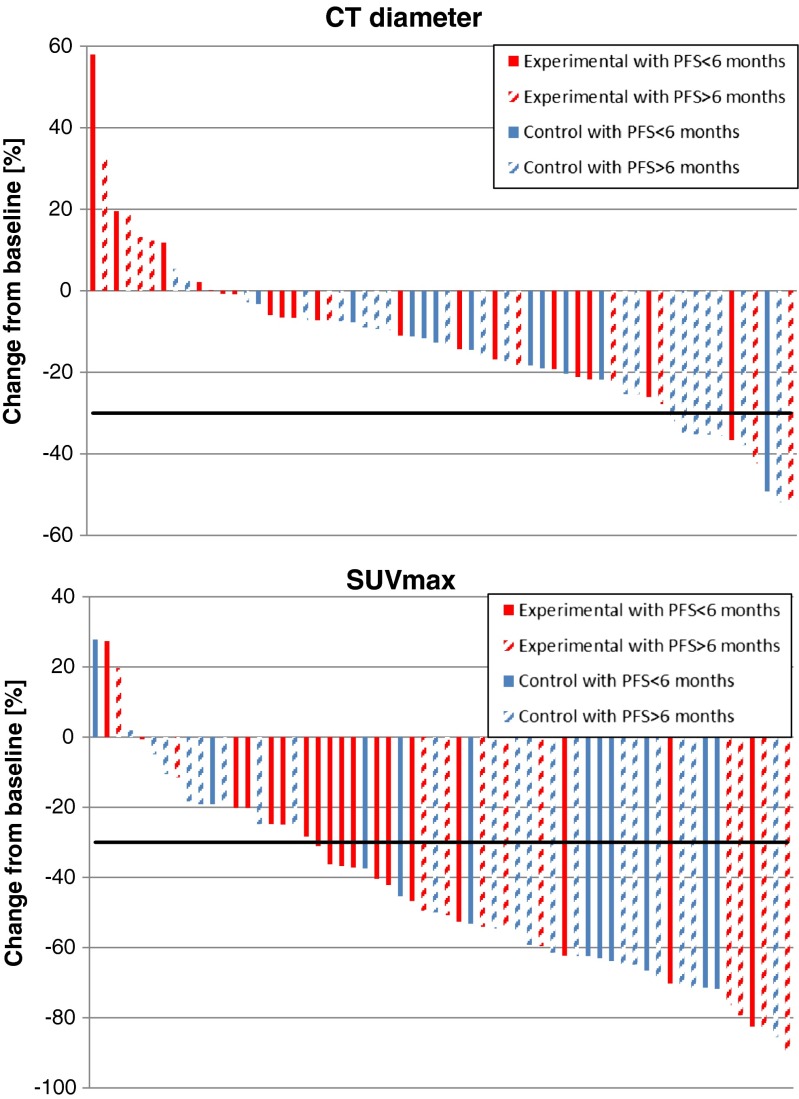
Change in CT diameter (upper) and SUVmax (lower) from baseline in individual patients. Patients of the experimental arm are plotted in *red*, patients of the control arm in *blue*. The *pattern-filled bars* represent patients with a progression free survival longer than 6 months. The *black line* represents the used response threshold of 30 %. SUV: standardized uptake value; PFS: progression-free survival

The changes in CT diameter and SUVmax between baseline and early response assessment were depicted in a waterfall plot showing that PET defined more patients as responders than CT (Fig. 4 and Table 2). However, this decline was not predictive for longer PFS (than 6 months).

## Discussion

The hypothesis of the NVALT12 trial was that the addition of NTG, by increasing tumor blood flow and oxygenation status, would improve outcome. While the clinical study of the NVALT12 already showed that NTG did not improve outcome, in the current study, we investigated if we could predict outcome based on early response assessment using [18F]FDG PET imaging [17]. This image analysis study of the NVALT12 trial could not show a predictive value of [18F]FDG PET imaging for the evaluation of the addition of NTG to bevacizumab-containing chemotherapy when compared to control patients. In a previous study, the administration of NO donating drugs decreased hypoxia-induced resistance to anticancer drugs in cancer cell lines [14]. In the NVALT12 trial, this could not be confirmed based on [18F]FDG PET analysis. This could be due to a lower NTG dose than that used in the Yasuda study [15], or to an interference with bevacizumab. From recent studies, it is known that FDG is only a moderate surrogate for hypoxia [32]. The study of Zegers et al. [33] showed that 42 ± 21 % of the primary tumor volume has a high FDG uptake (SUV > 50 % of SUVmax) of which 10 ± 12 % is hypoxic (high [18F]HX4 uptake TBR > 1.4), and that 3 % of the primary tumor volume outside the high FDG uptake volume is hypoxic as depicted by [18F]HX4 PET. In our study, we, therefore, only measure the effects of NTG on tumor metabolism and survival but not on hypoxia directly. Surprisingly, in nearly all patients, irrespective of treatment arm, a major decrease in FDG uptake was observed in the [18F]FDG PET scan performed after 3 weeks. Importantly, this [18F]FDG PET scan was acquired within 3 days after administration of the second cycle of chemotherapy. A study by van der Veldt et al. [34] showed that bevacizumab reduces tumor perfusion and [11C] docetaxel uptake in NSCLC, which was accompanied by rapid reduction in circulating levels of VEGF. This decrease in tumor blood flow after bevacizumab administration may explain the lower uptake of FDG in the tumor. Consequently, our results do not exclude the possibility that NTG decreases hypoxia.

A number of studies have demonstrated that changes in SUV parameters as early as the third week after the start of treatment are predictive for response to chemotherapy and PFS [1, 23, 24, 35]. A variety of approaches have been developed to measure the response, starting with the World Health Organization (WHO) criteria and continuing to RECIST and RECIST 1.1 [20, 26, 36]. These criteria refer to an anatomical decrease in tumor diameter. However, this response must be viewed with some caution when one is trying to predict outcomes in therapies that may be more cytostatic than cytotoxic. With such therapies, lack of progression may be associated with a good improvement in outcome, even in the absence of major shrinkage of tumors [37]. Newer metrics such as PET may be more informative [38]. PET/CT-based response evaluation has proven to be valuable in chemotherapy [39]. Currently, two sets of treatment response criteria for PET are available: EORTC and PET response criteria in solid tumors (PERCIST) [30]. PERCIST operates with a fixed ROI of 1 cm^3^ in the most [18F]FDG-avid part of the single most metabolically active tumor in the patient at each PET/CT scan. In the current study, a specific ROI, defining the primary tumor, was used for response evaluation. A consideration for anatomic and functional imaging is that many of the changes in response are at the border zones between response groups.

These border zones are quite artificial, as changes in tumor size are on a continuous scale (Fig. 3). The comparison of 30 % CT-based and 30 % PET-based response assessment performed after 3 weeks (median time interval between scans 42 days) with the RECIST analysis performed in week 6 (median time interval between scans 56 days) showed that the RECIST analysis defined more patients as responders than the 30 % CT-based analysis performed after 3 weeks. This can be caused by the difference in timing but also due to the fact that for the 30 % CT-based analysis, only one lesion was measured while in RECIST, multiple lesions were measured. The 30 % PET-based response assessment performed after 3 weeks showed more responders than the RECIST analysis, which is probably caused by decreased perfusion due to the bevacizumab treatment, which led to a decrease in FDG uptake for both treatment arms. The response assessment for PET was not influenced by NTG. A previous study of our group showed that after 3 weeks of treatment, five of nine patients were classified as responder by CT while six of nine were classified as responders by [18F]FDG PET [40]. In the same study, patients with a metabolic response (decrease in SUV > 20 %) at week 3 had a longer PFS than those without (9.7 months vs. 2.8 months), while patients with a response on CT at week 3 did not have a significantly longer PFS than those without. These two findings combined showed that PET may be able to show treatment response earlier than CT. In the former study, [18F]FDG PET scans were performed before bevacizumab infusion, while in our study the [18F]FDG PET scan was performed shortly after bevacizumab infusion. This might have impacted the uptake of FDG. A study of Hoekstra et al. [41] also shows that [18F]FDG PET has additional value over conventional radiologic techniques for monitoring response in locally advanced NSCLC patients.

The scans used for this study were made within the scope of the Dutch multicenter NVALT12 phase II trial and 60/223 patients underwent 2 [18F]FDG PET scans with the second scan after cycle 2, but before day 35. For quality control purposes, only scans with a mean SUV in the liver between 1.3 and 3 were used, reducing the number of assessed patients in this analysis to only 60 of the 223 original patients. For the analysis, these 60 patients were also divided between the control and the experimental arm, which means the study cohort was limited in size, hampering in-depth subgroup analyses.

## Conclusion

The addition of NTG did not lead to enhanced reduction in FDG uptake compared to the control arm. Although PET-based response assessment identified more responders than CT-based response assessment, this did not correlate to progression-free survival or overall survival. This might be due to the timing of the [18F]FDG PET shortly after the bevacizumab infusion.
